# Effect of Ball Screen and One-on-One on the Level of Opposition and Effectiveness of Shots in the ACB

**DOI:** 10.3390/ijerph18052676

**Published:** 2021-03-07

**Authors:** Jorge Serna, Verónica Muñoz-Arroyave, Jaume March-Llanes, M. Teresa Anguera, Queralt Prat, Aaron Rillo-Albert, David Falcón, Pere Lavega-Burgués

**Affiliations:** 1 Motor Action Research Group (GIAM), INDEST, National Institute of Physical Education of Catalonia (INEFC), University of Lleida (UdL), 25192 Lleida, Spain; vmunoz@gencat.cat (V.M.-A.); qprat@inefc.es (Q.P.); plavega@inefc.es (P.L.-B.); 2 Gens i Ambient en la Comprensió de la Diversitat de la Conducta Human i la Etiopatogénia del Transtorns Mentals (NeuroPGA), Faculty of Psychology, University of Lleida (UdL), 25003 Lleida, Spain; jmarch@pip.udl.cat; 3 Faculty of Psychology, Institute of Neurosciences, University of Barcelona (UB), 08007 Barcelona, Spain; tanguera@ub.edu; 4 Motor Action Research Group (GIAM), INDEST, National Institute of Physical Education of Catalonia (INEFC), University of Barcelona (UB), 08007 Barcelona, Spain; arilloalbert@gmail.com; 5 Motor Action Research Group (GIAM), INDEST, Grupo de Investigación en Entrenamiento, Actividad Física y Rendimiento Deportivo (ENFYRED), Facultad de Ciencias de la Salud y el Deporte, Universidad de Zaragoza, 50009 Zaragoza, Spain; dfalcon@unizar.es

**Keywords:** basketball, coach’s strategies, analysis performance, game actions, observational methodology, teamwork, effectiveness, offensive system

## Abstract

In light of the importance of shots on the final result in basketball matches, this research aims to reveal the predictive ability of variables associated with different game actions (ball screen, one-on-one near the basket and one-on-one far away from the basket), shot zone (near to or far from the basket) and players’ position (outside or inside) of the success of shots and of the type of shot, defended or not defended, in three seasons (2014 to 2016) of the Copa del Rey in the ACB (Asociación de Clubes de Baloncesto) League in Spain. Observational methodology was used, and an ad hoc instrument was created using Lince software. The study was conducted on a total of 7695 game actions, of which an analysis of 877 shots was made using CHAID (Chi-squared Automatic Interaction Detection) classification trees. The results obtained in this research have led us to the following conclusions: (a) the most important variable in the success of shots is to find “open shots”, regardless of the game action, (b) one-on-one actions outside and inside the zone have a clear connection to the finishes of the plays and end up in defended shots, (c) shots by ball screen lead to more open shots than in one-on-one and (d) play with passes may facilitate to find “open shots”. These results can be of interest for coaches to prepare their teams in the best possible way, but they must be interpreted with caution as the sample was very specific (Copa del Rey in the ACB League) and, therefore, it is necessary to continue investigating this topic in other competition contexts.

## 1. Introduction

Victory or defeat in basketball, as in all team sports, depends on the success of the finishes; in the case of basketball, this is due to the effectiveness of shots at the basket [1,2,3]. Consequently, although basketball has traditionally been referred to as a cooperation–opposition sport, the dominant relationship on the game final outcome is opposition [2], for which reason it should more accurately be called an opposition–cooperation sport [4].

### 1.1. The Offensive Strategy: Defended and Not Defended Shots from Different Zones

To achieve success in finishing, basketball coaches design offensive strategies (OS) that are aimed at creating optimal shooting situations [4,5,6,7,8]. The term optimal shooting situations could be given to those situations that are executed by the best shooters in the contexts that best suit them or that display the best percentages of success [7,9]. The OS needs to consider different aspects [10,11], amongst which we can highlight two: (a) the level of opposition or defence that the shooter has when taking the shot [1,6,7,10,11], and (b) the distance of the shot from the basket [5,6,7,9,10]. Taking these variables into account, the first objective should be to find open shots close to the basket. The next one should be to find shots close to the basket with a low level of opposition (the shot is defended, but as it is close, the level of effectiveness is usually high) or, at a similar level of interest, open shots from further away. The final option, and one that is not particularly welcomed by coaches, would be to take defended shots far away from the basket [4,8,10,11]. It is true to say that occasionally, players that are more skilful at taking shots are capable of scoring, even in situations that are defended [9,12,13].

When the defensive team is disorganised or reorganising, shots at the basket are usually more successful, as is borne out in other studies [8,14,15,16]. These are situations of numerical superiority by the attackers where the defence is disorganised, situations of fast transition from one end of the court to the other where the defensive team is reorganising and, finally, situations after an offensive rebound [11,14] where the defence is not organised, which bear similarities to fast breaks [8,16].

Therefore, the OS will seek to increase the number of game situations of these characteristics, where the rival is not organised or is reorganising. The problem is that, nowadays, the defensive organisation of top-level teams is excellent, and it is very difficult to score these “easy” baskets [8,11,17]. For this reason, it seems necessary for coaches to have OS in order to get advantage in situations under numerical equality in half court, i.e., 5-on-5 [16,17,18,19]. This is an aspect that is notoriously difficult to finish with an offensive advantage as possession time is limited (24 s), the playing area is very small and the level of opposition in the duel is very high [11,12,16].

### 1.2. Game Actions for Taking Shots

In these situations of numerical equality, where the playing area is limited, time is very often against the team with the ball. The team needs to create advantages to be able to find these high-percentage shots [18]. This is why one of the main objectives of the OS in positional attacks will be to create advantageous situations. To this effect, coaches propose playing systems (sets) based on different game actions [20] according to whether the aim is to favour the actions of the ballhandler or the actions of the player without the ball [4]. This research will take an in-depth look at the actions linked to the ballhandler, such as ball screen (BS) and one-on-ones (1-on-1), distinguishing duels near the basket (1-on-1 inside) and duels far away from the basket (1-on-1 outside), also called “clear outs” [8,21,22].

BSs are key game actions in modern basketball due to their high frequency of use and high yield [17,23,24,25,26,27], and even for their possible direct influence on the game final outcome [28]. Some of the positives of the use of BSs are: (a) helping the ballhandler in their advance on the rival basket, (b) enabling a shot by the ballhandler with no defensive pressure, (c) passing to the screener who has moved towards the basket (known as Pick and Roll), (d) passing to the screener who has moved away from the basket (known as Pick and Pop), (e) passing to a third player who was not directly involved in the BS, (f) provoking defensive changes in the opposing team, creating mismatches, (g) maintaining the continuity of possession with the aim of continuing to look for definitive advantages and, finally, (h) looking for a quick finish when possession time runs out. This research will focus solely on the shot at basket by the ballhandler in the BS.

With regard to the 1-on-1 outside (1-on-1 Out) or “clear outs”, this is an action used frequently in positional situations, such as in final moments of possession where many attacks have to be resolved in this way. This game action calls for the ballhandler’s skill to resolve the duel between individuals [22,29]; very often, it is not just a confrontation between two players, as a pure 1-on-1 duel never occurs in basketball. There are generally teammates and/or opponents that affect the situation, removing the concept of the individual duel or at least reducing it within the collective scenario [4,16].

As borne out by other research [30,31,32], the 1-on-1 inside (1-on-1 In) has historically been one of the strategic cornerstones of coaches in their OS for different reasons: (a) it allows high-proximity shots, increasing the probability of success, (b) it takes the ball into a very dangerous place for the defending team, which has to make a decision about the opposing invasion, (c) it favours passes with another inside player, (d) it favours passes with outside players whose opponents have had to help too much, (e) it provokes fouls due to the risk of scoring that it entails and, finally, (f) it enables the subsequent offensive rebound in the event of not scoring. As was mentioned earlier, this research will focus solely on the shot at basket when playing the 1-on-1 In situation.

This research analysed the shots that originated from three game actions (1-on-1 Out, 1-on-1 In and BS) in positional offense against individual defences. In this study, these shots had to be by the player with the ball who started the action, i.e., what is known as the ballhandler. The moment the ballhandler passed the ball, they were no longer the subject of this research. This study is immersed in a line of research addressing what can be the most effective game actions to be proposed by the coach through his OS to achieve effectiveness in their attacks [8,21,33]. The main goal is to try to generate scientific knowledge to help coaches both to create their own strategies and to anticipate what can happen when the rival team plays a certain offensive game action (1-on-1 and BS).

In short, in light of the theoretical framework described, this research proposed two objectives referring to the matches in three seasons of the Copa del Rey of the ACB League (2014, 2015 and 2016):

(a) To reveal the predictive ability of variables associated with different types of actions in which the shot originates (BS, 1-on-1 In, 1-on-1 Out), level of opposition in the shot (defended or not defended), shot zone (six zones) and players’ position (outside or inside), of the success of the shots.
(b) To reveal the predictive ability of variables associated with different types of actions in which the shot originates (BS, 1-on-1 In, 1-on-1 Out), shot zone (six zones) and players’ position (outside or inside), of defended or open shots.

## 2. Method

### 2.1. Research Design

We employed Observational Methodology [34] which has proven to be a robust scientific method for analysing complex games such as basketball [8,30,35]. It is a direct and non-participating observation study. The observational design used was N/F/M [36,37]: nomothetic (N), because various matches were observed, follow-up (F), because various matches were examined over three high-level men’s championships, and multidimensional (M), as different dimensions were considered, on the basis of which the observation instrument was built.

### 2.2. Participants

Three basketball Copa del Rey were analysed, which took place in 2014, 2015 and 2016 in the cities of Málaga, Las Palmas de Gran Canaria and A Coruña (Spain), respectively. A total of 21 matches (7 each year), 12 teams and 7695 actions were observed, of which 2041 finishes in positional attack and individual defence were analysed (648 in 2014, 686 in 2015 and 707 in 2016). The teams analysed are shown in Table 1.

### 2.3. Observation Instrument and Procedure

The matches broadcast by a national television channel were recorded on video files in AVI (Audio Video Interleave) format. These video files were then coded using Lince v. 1.3 software [38], which enabled us to have a record in the form of code matrices, such that each row in the matrix showed the co-occurrences of behaviour that had occurred between the different dimensions or criteria. The data obtained are type IV (concurrent/base time) [39], although they have been analysed omitting the duration information, such as type II data (concurrent/base event). For the statistical analysis, the data recorded were exported in Excel 2013 (Microsoft Corporation, Redmond, WA, USA).

The observation instrument used was a simplified version of the Key Concepts in Basketball Observation System (SOCCB), validated by Reference [33]. This instrument is comprised of a total of 7 criteria and 23 categories (Table 2).

To code the data, an observer manual was prepared, describing the criteria, the categories and the respective degree of freedom of the categories. The observational register was made by a basketball coach with a degree in Physical Activity and Sport Sciences, with more than ten years of coaching experience and experience in basketball research using Observational Methodology. The observer was coached in following the instructions in the manual carefully.

### 2.4. Data Quality Control

Analysis of the data quality control was carried out with 30% of the attacking sequences of the Copa del Rey 2014. To verify the intra-observer reliability, Generalizability theory (G theory) and instrument homogeneity were applied using a two-facet design (categories/observers = C/O and observer/categories = O/C, respectively) with SAGT v1.0 software [40,41]. The estimation of the variance components was carried out infinite-randomly, both for the categories facet and for the observer facet. This analysis revealed that most of the variability was associated with the categories facet (99.96%) and was nil in the observer facet and very low in the categories/observers interaction facet (0.05%). The analysis of the generalizability coefficients indicated a high reliability of generalization accuracy of the results of 1.

The Cohen kappa concordance coefficient was also calculated using IBM SPSS Statistics v. 23.0 software (SPSS Inc., Chicago, IL, USA). Values higher than or equal to 0.93 were obtained in all the observation instrument criteria. Therefore, in all the Cohen kappa coefficients calculated, an almost perfect concordance force was obtained [42].

**Table 2:** SOCCB criteria and categories.

Criteria	Categories (Codes)	Definition
Game actions	Ball screen (BS)	Screen performed to the player with the ball, so that he can get rid of his defender
	One-on-one outside (1-on-1 Out)	Player with the ball attacks against his rival from out of the three points line
	One-on-one inside (1-on-1 In)	Player with the ball attacks against his rival close to the paint (restricted area with the 3-second rule)
Offense type	Fast break (FB)	Fast offense with numerical superiority
	Transition (TR)	High-tempo offense in numerical equality
	Positional Offense (PO)	Positional or static 5-on-5 in half court
Defence type	Individual (IND)	Each player of the defensive team guards a single player on offense
	Zone (ZON)	Each defensive player is given an area of the court to cover. Different options: 2-3, 3-2, 1-3-1
	Mixed (MIX)	Some players defend in zone, some in man-to-man. Different options: Box and one, Triangle and two
	Unknown (UNK)	It is not possible to identify the type of defence used
Player position	Outside player (O)	Point Guard, Guard, Forward
	Inside player (I)	Power Forward, Centre
Shot zone (Figure 1)	End Zone 1 (EZ1), End Zone 2 (EZ2), End Zone 3 (EZ3), End Zone 4 (EZ4), End Zone 5 (EZ5), End Zone 6 (EZ6), End Zone 7 (EZ7)	Shooting zone. It is the zone of the court, as shown in Figure 1, from which the shot is executed.
End of offense	Successful shot (SUC)	When the shooter scores or gets a foul
	Unsuccessful shot (UNS)	When the shooter misses or does not get a foul
Level of opposition	Defended (DEF)	Contested shot: The shot is taken with an opponent in front of the shooter
	Not Defended (NDEF)	Uncontested shot: The shot is taken without an opponent in front of the shooter

### 2.5. Data Analysis

First of all, a descriptive analysis of the game actions was conducted (BS, 1-on-1 Out and 1-on-1 In) on the success of the defended and open shots, in positional offenses against individual defences. Subsequently, the normality of the data was examined using the Kolmogorov–Smirnov test, and the normality hypothesis was rejected due to the asymmetry of the distribution. Therefore, the CHAID classification trees technique was applied [43], using a cross-validation system with a rule of detention of three maximum levels, with 50 being the minimum number of cases in the terminal nodes.

A CHAID classification tree was created using different predictive variables: (a) actions in which the shot originates (1-on-1 Out, 1-on-1 In, BS), (b) the level of opposition (defended/not defended), (c) the success of the finish (successful/unsuccessful), (d) the shot finish zone and (e) the player’s position (inside/outside), to determine the success or ineffectiveness of the shots. These predictive variables were also examined on opposed and unopposed shots. Both analyses were made on IBM SPSS Statistics v. 23.0 software (SPSS Inc., Chicago, IL, USA), grouping the data of the three competitions analysed and setting a significance level in the second analysis of *p <* 0.05.

## 3. Results

A total of 7695 actions in the 2014, 2015 and 2016 Copa del Rey were recorded, including pass and finish actions in fast breaks, transitions and positional offenses. Out of the total sample, 2041 actions that finished in a shot using positional offense and against an individual defence were analysed. Of these actions, 42.96% (877) saw the use of 1-on-1 Out, 1-on-1 In or BS (Ball Screen) as actions that originated the shot.

By analysing the use of 1-on-1 Out, 1-on-1 In or BS actions, it was observed that the principal consequence of the 1-on-1 Out (61.2%) and of the 1-on-1 In (67.8%) were shots, whereas the principal consequence of the BS were passes (73.6%).

### 3.1. Predictive Variables of Success or Failure in Shots

**Figure 2:**
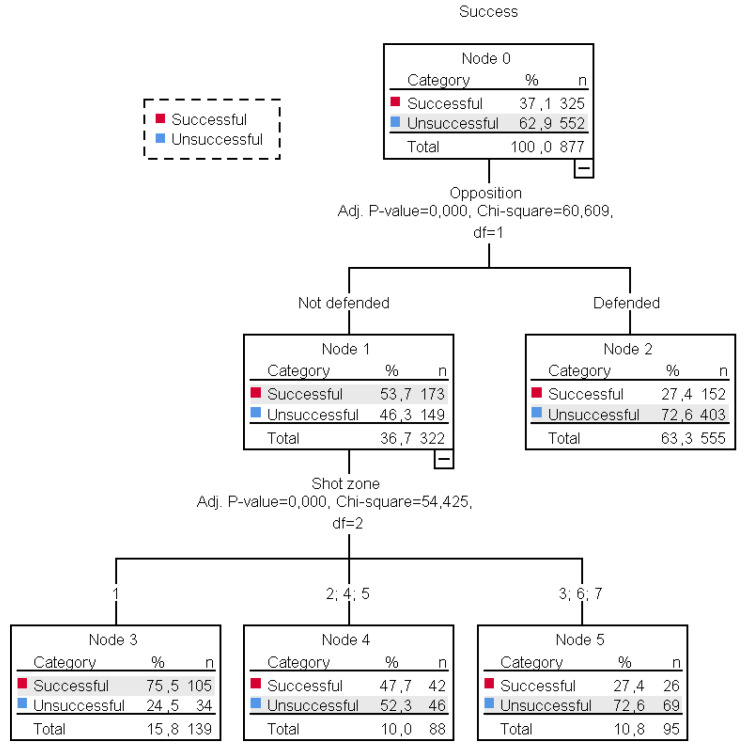
Level of opposition and shot zone as key variables in predicting shot success.

A CHAID classification tree was created to study the predictive ability of the variables: (a) actions in which the shot originates, (b) the level of opposition (defended/not defended), (c) the success of the shot (successful/unsuccessful), (d) the shot zone and (e) the player’s position (inside/outside), on the success of the shots (Figure 2).

Six nodes were identified to analyse 877 shot actions, of which 552 were unsuccessful shots and 325 were successful shots.

The classification tree identified the level of opposition (defended/not defended) as the first predictive variable (nodes 1 and 2), i.e., if the shooter was open or defended. Significant differences were observed (*x*^2^ (1) = 60.609, *p* < 0.001) between the not defended shots (36.7%, success = 53.7%) and the defended shots (63.3%, success =27.4%).

The second predictive variable for the success of the not defended shots was the shot zone (nodes 3, 4 and 5), (*x*^2^ (2) = 54.425, *p* < 0.001). The majority of not defended shots were taken from zone 1 (*n* = 139, 15.8%), recording the highest percentage of success (75.5%). Success in zones 2, 4 and 5 was similar to shots missed (success: *n* = 42, 47.7%; unsuccessful: *n* = 46, 52.3%). Successful shots reached the lowest values in zones 3, 6 and 7 (*n* = 69, 72.6%).

No predictive variables were recorded for defended shots (node 2).

### 3.2. Influence of 1-on-1 and BS on Shots

**Figure 3:**
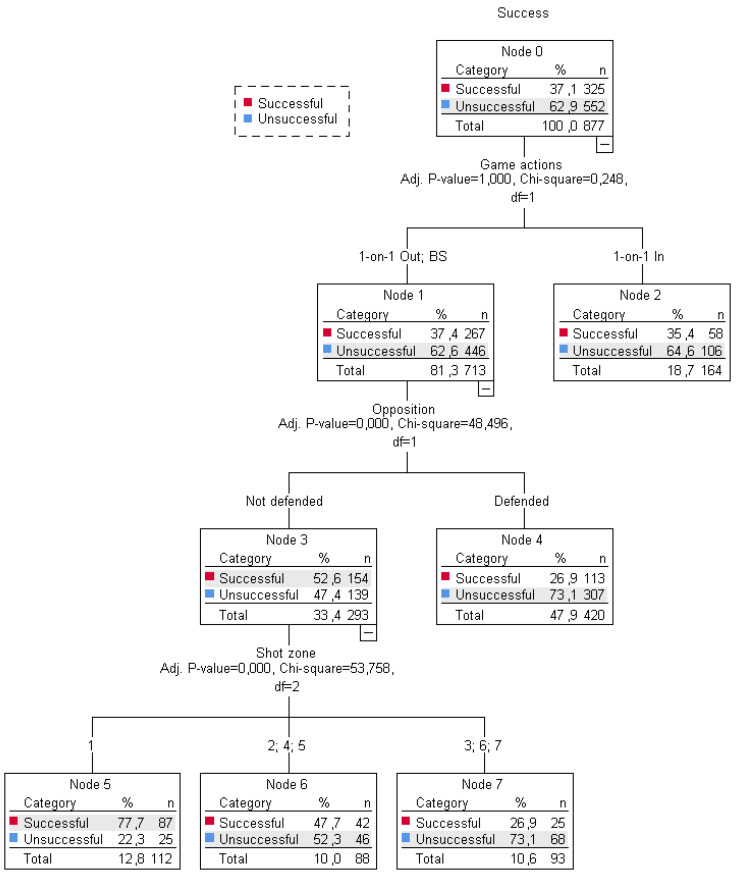
Game actions (1-on-1, Ball Screen) and shot zone as key variables in predicting shot success.

Once the results of the first classification tree were analysed, it was decided to apply a second classification tree, incorporating a new predictive variable: game actions (1-on-1 Out, 1-on-1 In and BS). The CHAID procedure allows these types of restrictions to be introduced. A tree with 8 nodes was created (Figure 3).

The game actions were identified as the first predictive variable (nodes 1 and 2). Besides recording similar percentages in the different game actions, no significant differences were observed (*x*^2^ (1) = 0.248, *p* = 1) between the 713 shots (81.3%) taken with 1-on-1 Out or BS actions (node 1) (successful: *n* = 267, 37.4%; unsuccessful: *n* = 446, 62.6%) and the 164 shots (18.7%) with 1-on-1 In actions (node 2) (successful: *n* = 58, 35.4%; unsuccessful: *n* = 106, 64.6%).

Shots taken with 1-on-1 In actions did not have more predictive variables. For the rest of the shots with 1-on-1 Out or BS actions, the following explanatory variable was opposition. Based on this variable, a similar behaviour to the first tree was observed in the variables involved (opposition and shot zone) and the successful and unsuccessful shot percentages.

### 3.3. Predictive Variables of Defended or Not Defended Shots

**Figure 4:**
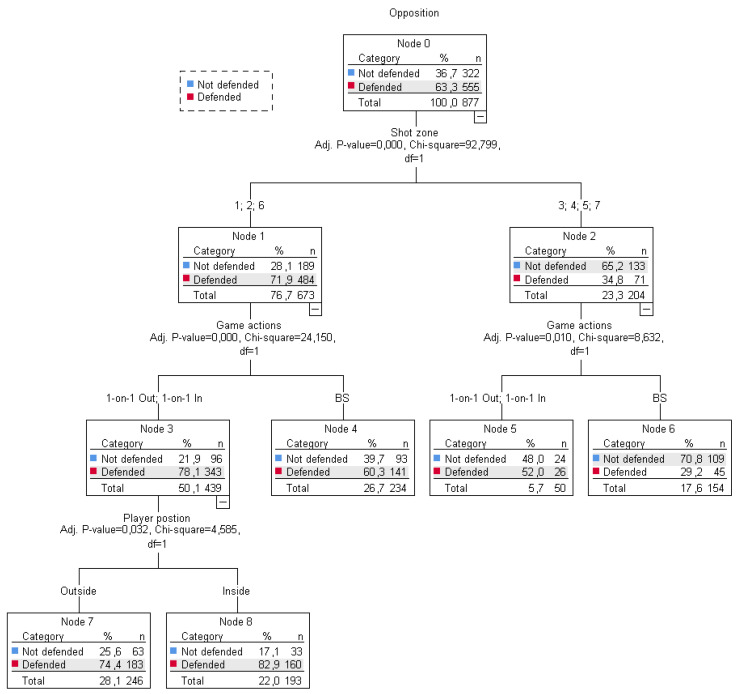
Shot zone, game actions (1-on-1, BS) and player position as predictive variables in the level of opposition in the shot.

The tree in Figure 4 was used to analyse the predictive strength of the variables: shot zone, game actions in which the shot originates and players’ positions, on the 877 defended shots (*n* = 555, 63.3%) or not defended shots taken (*n* = 555, 36.7%).

The first predictive variable was the shot zone. Significant differences (*x*^2^ (1) = 92.799, *p* = 0.001) were found between shots taken in two groups of zones 1, 2 and 6 (node 1) (not defended: *n* = 189; 28.1%; defended: *n* = 484; 71.9.1%) and zones 3, 4, 5 and 7 (node 2) (not defended: *n* = 133, 65.2%; defended: *n* = 71, 34.8%).

The next predictive variable for shots in zones 1, 2 and 6 was the game actions. Significant differences (*x*^2^ (1) = 24.150, *p* < 0.001) were found between shots taken using 1-on-1 Out and 1-on-1 In actions (node 3) (not defended: *n* = 96, 21.9%; defended: *n* = 343, 78.1%) and those taken by BS (node 3) (not defended: *n* = 93, 39.7%; defended: *n* = 141, 60.3%).

In this part of the tree, the players’ position was identified as the predictive variable of shots using 1-on-1 Out and 1-on-1 In actions. Significant differences (*x*^2^ (1) = 4.585, *p* < 0.032) were found between shots taken from the outside position (node 7) (not defended: *n* = 63, 25.6%; defended *n* = 183, 74.4%;) and shots from the inside position (node 2) (not defended: *n* = 33, 17.1%; defended: *n* = 160, 62.9%). Shots in zones 1, 2 and 6 with BS actions did not have more predictive variables.

On the right of the tree, corresponding to shots from zones 3, 4, 5 and 7, game actions were also identified as a predictive variable. However, the behaviour of the defended and open shots for the same variables was different to that observed in the other zones 1, 2 and 6. Significant differences (*x*^2^ (1) = 8.632, *p* < 0.001) were found between shots taken using 1-on-1 Out and 1-on-1 In actions (node 5) (not defended: *n* = 24, 48%; defended: *n* = 52, 26%) and those taken by BS (node 6) (not defended: *n* = 109, 70.8%; defended: *n* = 45, 29.2%).

### 3.4. Influence of 1-on-1 and BS on the Level of Opposition in Shots

**Figure 5:**
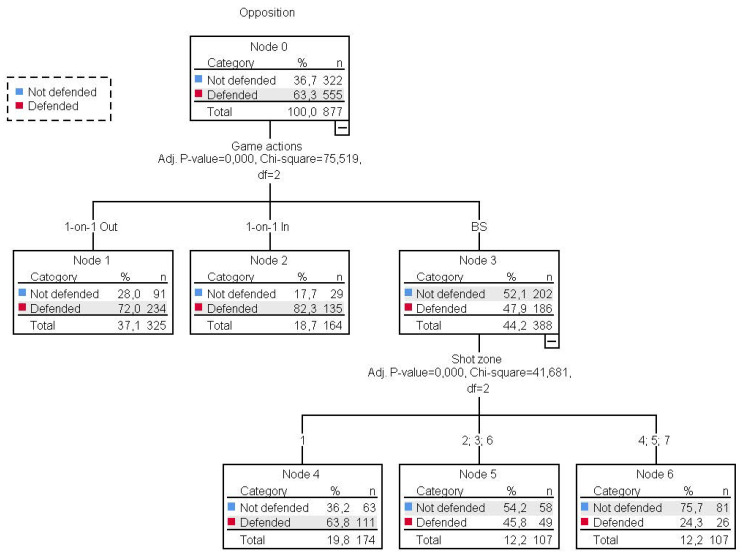
Game actions (1-on-1, Ball Screen) and shot zone as predictive variables in the level of opposition.

To take a more in-depth look and complement the results of the previous classification tree, it was decided to apply the same statistical technique, incorporating a new predictive variable: game actions (1-on-1 Out, 1-on-1 In and BS). Remember that the CHAID procedure allows these types of restrictions to be introduced. A tree with 6 nodes was created (Figure 5).

The tree identified two predictive variables: the game actions and the zone from where the shots were taken.

The game actions were the first predictive variable of the defended and not defended shots. Significant differences were found between the three types of action (*x*^2^ (2) = 75.519, *p* < 0.001). The majority of shots (*n* = 44.2%) were taken using BS actions, displaying a similar proportion between the not defended shots (*n* = 202, 52.1%) and the defended shots (*n* = 186, 47.9%). The not defended shots were appreciably lower than the defended shots when they were taken using 1-on-1 Out actions (node 1, not defended: *n* = 91, 28%; defended: *n* = 234, 72%) and 1-on-1 In actions (node 2, not defended: *n* = 29, 17.7%; defended: *n* = 135, 82.3%).

The tree identified a second predictive variable, the shot zone, although only to explain the actions taken using BS. Significant differences (*x*^2^ (2) = 41.681, *p* < 0.001) were found between zones 1 (node 4), zones 2, 3 and 6 (node 5) and zones 4, 5 and 7 (node 6). The largest quantity of shots using BS were recorded in zone 1 (*n* = 174, 19.8%), where there was a greater superiority between defended shots (*n* = 111, 63.8%) and not defended shots (*n* = 63, 36.2%). This behaviour was inverted in shots using BS in zones 4, 5 and 7 (defended: *n* = 26, 24.3%; not defended: *n* = 81, 75.7%). The differences were not as accentuated in shots from zones 2, 3 and 6 (defended: *n* = 49, 45.8%; not defended: *n* = 58, 54.2%).

## 4. Discussion

This research proposed the objectives of revealing the predictive ability of variables associated with different game actions (BS, 1-on-1 In, 1-on-1 Out), shot zone (seven zones) and players’ position (outside or inside) of the success of shots and also of the type of shot, defended or not defended, in three seasons of the Copa del Rey in the ACB League (2014, 2015 and 2016).

One, first, has to bear in mind that our research deals with a specific and small sample of the ACB League due to the choice of a short competition such as the Copa del Rey ACB during 3 successive seasons (2014, 2015, 2016), with a total of 21 games. Consequently, interpretation of these data will take into account this very specific context

### 4.1. The Key To a Successful Shot: The Search for Not Defended Shots

The first finding of the research identified the open (not defended) shot at basket as the first predictive variable of success in positional 5-on-5 situations against an individual defence, as reported by other research in other contexts [7,10,12,35]. These data would allow us to think about the fact that, if the coach’s OS encourages cooperation strategies among the players of his team, it can favour the obtaining of open shots (not contested) [4,8], which means that playing with passes could help with being more effective in possessing, as other investigations in different contexts already stated [8,11,16,30].

It was also observed that, in this specific competition, not defended shots were much lower in number than defended shots. This fact together with the contributions of other researches in other competitions [14,44,45] encouraged us to interpret that, in recent years, defensive performance in professional teams seems to be improving for several possible reasons: (a) the coach’s defensive strategy proposes a network of collaborations between the players in the team that prevents pure 1-on-1 duels and not defended shots [4,8,11,44], (b) scouting or studying the strengths and weaknesses of the opposing team enables the adjustment of teams’ defensive strategies to the peculiarities of the opposing players who will be playing in the next match [45,46] and (c) by having improved their technical, tactical and physical performance, today’s players can defend their opponent’s attacks with greater intensity and intelligence [47,48,49].

As a consequence of these arguments, some researchers [14,15,16] suggest that a way to make possessions more effective consists of attacking when the defensive team is not well organized, such as fast breaks, transition game or after an offensive rebound. One could interpret that when defence is not well located, it could be easier either to go to the rival’s basket for a close shot or to find comfortable passes as receivers are not under pressure and, that way, there could be open players (without near opposition) with time to shoot with certain ease [7,10,12,16]. The data obtained via this investigation contribute to the idea that it is hard to find open shots playing 5-on-5 half-court regardless of the game action used [4,8,33].

On the other hand, it has been observed that 27.4% of successful shots are taken defended. This percentage is not to be dismissed and confirms the great skill of players in scoring shots in very difficult situations [7,9,22]. In addition, when these shots are taken in zones near the basket, they are often in the face of the opponent’s defence (e.g., 1-on-1 Out situations) [8,21,30,31].

These data would demonstrate the importance of training in opposed shot-making in dribble actions (pull-ups), both in BS and in 1-on-1 Out and 1-on-1 In, providing the player with an extensive repertoire based on their ability to score in more difficult situations [7,9,13,22]. It also confirms once again the role of the opposition in this sport, as despite not obtaining team advantages, very often, individual effectiveness can achieve the desired objective [4,8,13].

### 4.2. Shooting Space: Effectiveness and Gaining an Advantage

In order to have a correct interpretation of the data, it is necessary to remember that this study is not conducted on shots that come from passes, but on shots executed by the player with the ball when starting the one-and-one and BS.

The data obtained in this research reveal that, in the considered context (Copa del Rey ACB), the shooting space was a key variable that can help with predicting the success of a shot, providing it is taken not defended (open shot). An interesting finding of this investigation is that as far as defended (contested) shots are concerned, the zone from where the shot is taken does not matter, as this action will be taken under adverse conditions and, consequently, with a very low level of success [12,14,44].

The findings in this tournament show that the best performances when executing these game actions (1-on-1 and BS) were obtained with not defended shots close to the basket, confirming conclusions from other studies [5,10,32,35], and, if close shots were not possible, they tried to go for not defended shots towards the centre of the court, as suggested by other researches analysing finishes in carrying the ball in these kinds of situations [8,26,27,28]. In the same line, it is normal that the spaces towards the corners are not very frequent. This is due to the fact that the player with the ball will look for spaces that attack the centre of the defensive court, where the opposing basket is situated [11,29,30,31].

On the other side, the defensive team will react by accumulating opponents in order to protect their space, trying to make the ballhandler pass the ball or take a shot under unfavourable conditions [11,50,51]. So, data point out that, due to the defensive response of the opponent, on many occasions, the player has to pass the ball to other teammates who are free, as mentioned in the literature [7,8,10,35], situated in the free spaces outside the 3-point line, on the sides and at the corners. Therefore, researchers confirm the need to improve the success percentages in these zones, as they are shots that are not defended or are less defended than those closest to the basket [10,16,32,45].

Therefore, it is a constant battle for space between the team with the ball and the team without the ball. The team with the ball wants to reach the desired space, the hoop, while the team without the ball uses its strategies to prevent its rival from achieving its desires. In conclusion, it is a duel between teams with the objective of dominating the space [11,50,51].

The data obtained (Figure 4) show that, when players started a 1-on-1 Out, they mostly tried to get close to the basket. Only 50 times in 21 matches did they try an outside shot. In other words, the pull-up action, as shown by other research [52,53], is a scarcely used action in the outside space but one that permits a low level of opposition (48% in zones 3, 4, 5, 7) compared with other shots. When players play a 1-on-1 Out, they want to go towards the basket and they very often come up against opponents and take the shot opposed. Talking about BS, they get a high percentage of free shots (39.7% in zones 1, 2, 6; 70.8% in zones 3, 4, 5, 7). More specifically, we can see in Figure 5 that when playing BS, the defending pressure on shots decreases as the shot gets further away from the basket. Therefore, besides being a building procedure, BS also allows the ballhandler to shoot under better conditions than by using 1-on-1 actions [8,26,53]. Consequently, it would be interesting to develop programs to improve efficiency in this kind of shot that is free but far from the basket [6,13,35,54].

### 4.3. Effect of 1-on-1 and BS on Offensive Performance

The findings of this research are in line with the evidence found in other studies [8,21,23,25,53] and can be summarised in three sections: (a) the BS has been the most widely used action, which is confirmed by its importance in modern basketball, and (b) the most frequent consequence of playing BS (73.6%) is passing the ball. This is a procedure aimed at collaborative game-building as teammates cooperate to help the player be able to take a successful open shot. (c) 1-on-1 Out duel actions feature more than 1-on-1 In actions. This is probably due to the spatial limitations close to the basket.

Another finding of this research is knowing that 43% of the possessions analysed finished with a shot with BS or 1-on-1 actions. These are shots taken in positional offense and in individual defence, which are highly influential on offensive performance in basketball, as has been shown by other studies [8,26,31,53].

It has been observed that there are significant differences between shots taken from originating outside (1-on-1 Out and BS) and originating inside (1-on-1 In). It is observed that both shots that are preceded by actions originating outside (1-on-1 Out and BS) and those that have as origin the 1-on-1 In have a similar success (35%), which could be considered as low performance, according to the findings of other studies [10,26,55].

In light of this finding, it is suggested that this kind of action based on individual offenses without passes would not be the most effective in this type of competition and that it would likely be better to find offenses with passes, where the aim is to try to find shots under better conditions [11,16,45,53]. Hereafter, we present a deeper analysis of these data: shots originated inside, and shots originated outside (BS and 1-on-1 Out).

With regards to the impact of 1-on-1 In on this specific sample, which obviously cannot be generalizable to all situations in basketball but only for this particular context, we come to the following conclusions, which are also in line with other previous contributions [8,21]: (a) slight use of this game action (*n* = 164) compared to game actions originated outside (*n* = 713), (b) 1-on-1 In has a clear aim of shooting, as little more than 30% of 1-on-1 In actions end in passes, (c) it is an easily executed shot near the basket, hence a positive action, (d) it is an action that is usually taken against the demanding defence of an opponent of similar morphological and strength characteristics and, finally, (e) the effectiveness of these shots is not very high (35%).

It is true that this 1-on-1 In can be played by outside players [24] but, in this research, the result of this game action was similar: one-and-one with the goal to score higher than to pass, and this shot will be, the majority of times, defended. Therefore, we are presented with a game action that has to be explored in other competition contexts because some studies propose this procedure to obtain success [30,31], whereas the above-mentioned studies [8,21] show that 1-on-1 In is increasingly more of a finishing procedure than one of collective game building. These reflections lead us to believe that, as in all game actions, the performance will depend on the kind of players that execute them. It will be very difficult to find absolute truths when studying this kind of game actions, rather trends.

As far as the impact of the actions originated in the outside play (BS and 1-on-1 Out) is concerned, this research found no differences in the effect on the shot success between playing BS or 1-on-1 Out. It has been commented earlier that BS was an action aimed at building (passes) [8,21], but these data (with this limited sample) confirm that once the decision to shoot using BS has been made, it behaves in a similar way to 1-on-1 Out.

However, the differences are significant when comparing shots that are taken defended or not defended according to game actions. By playing BS, not defended shots are created in half of the cases. By playing BS and trying to finish close to the basket (zone 1), almost 64% of the shots were obviously defended, but when shooting from middle range with dribble (pull-ups), and more than 50% of three-pointers pull-up too, in both cases, they released shots. These data reinforce the findings obtained in other research which showed important advantages of the ballhandler when playing BS and the need of improving effectiveness in shots generated by situations [27,28,35,53].

By contrast, both inside and outside 1-on-1 are practically synonymous with defended shots, especially with 1-on-1 In (82.3% of defended shots), but also with 1-on-1 Out (72%). As commented earlier, it is difficult to find clear advantages that result in not defended shots from using 1-on-1 situations because it is not a pure 1-on-1 as the defender is usually helped by their teammates [8,11,44]. So, as mentioned in other research [26,28], BS has to be also considered as a game action, which is interesting to find the ballhandler’s score.

This finding is of great interest when considering strategies that look for not defended shots. In line with other studies [8,11,16], if the aim is to find not defended shots, it would be interesting to work on cooperation between teammates (BS) rather than looking for the 1-on-1 duel. It is true that this assertion should be made cautiously if we consider the level of effectiveness of shots from BS and 1-on-1 in this research. As a result, it would be advisable to work on improving the effectiveness of not defended shots using BS situations, for example pull-ups or other kind of shooting actions [27,28,53,56].

## 5. Conclusions

Our research deals with a specific and small sample of the ACB League since we have only studied three editions of the Copa del Rey, which means 21 games. So, we are careful to give recommendations for practical use. It would be of interest to continue with this line of research in other competition contexts to know if these results can be extrapolated. In any case, these contributions can be useful for coaches in order to prepare their teams in the best possible way, both from an offensive and from a defensive point of view.

The most important conclusions of the research were: (a) the main variable for the success of shots was to find “open shots”, regardless of the “game action” used, (b) one-on-one actions outside and inside the zone have a clear connection to the finishes of the plays and end up in defended shots, (c) shots by ball screen lead to more open shots than in one-on-one, (d) the player who shoots in BS situations obtains not defended shots, but does not have a greater effectiveness than ones made using 1-on-1 Outside and (e) one of the bases to improve effectiveness in possessions is to increase the shooting effectiveness in multiple situations (not defended, defended, playing one-and-one, BS, amongst others).

## Figures and Tables

**Figure 1:**
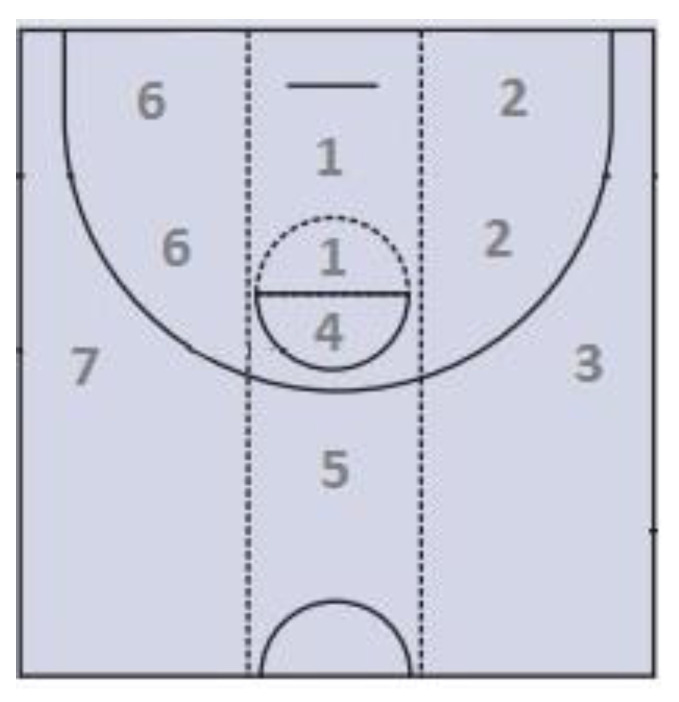
Shot zone.

**Table 1:** Participating teams in the 2014, 2015 and 2016.

Team	Matches Played per Team during the Copa del Rey		
	2014	2015	2016
Bilbao Basket		1	2
Basket Zaragoza	2	1	
F.C. Barcelona	3	3	1
Joventut de Badalona		2	
C.B. Gran Canaria	1	1	3
C.B. 1939 Canarias (Tenerife)	1		
Saski Baskonia S.A.D.	1		2
Real Madrid	3	3	3
Baloncesto Málaga	1	2	
Valencia Basket	2	1	1
B. Fuenlabrada S.A.D.			1
Obradoiro C.A.B. S.A.D.			1

## Data Availability

Not applicable.
